# KRAS, MYC, and ARF6: inseparable relationships cooperatively promote cancer malignancy and immune evasion

**DOI:** 10.1186/s12964-023-01130-3

**Published:** 2023-05-08

**Authors:** Hisataka Sabe

**Affiliations:** Department of Molecular Biology, Graduate School of Medicine, and Institute for Genetic Medicine, Hokkaido University, Sapporo, Japan

**Keywords:** AMAP1, ARF6, Cancer immune evasion, Cancer malignancy, eIF4A, KRAS, MYC, Mitochondria, mTOR, G-quadruplex structure, TP53

## Abstract

Mutations in the *KRAS* gene and overexpression of protein products of the *MYC* and *ARF6* genes occur frequently in cancer. Here, the inseparable relationships and cooperation of the protein products of these three genes in cancer malignancy and immune evasion are discussed. mRNAs encoded by these genes share the common feature of a G-quadruplex structure, which directs them to be robustly expressed when cellular energy production is increased. These three proteins are also functionally inseparable from each other, as follows.　1) KRAS induces *MYC* gene expression, and may also promote eIF4A-dependent *MYC* and *ARF6* mRNA translation, 2) MYC induces the expression of genes involved in mitochondrial biogenesis and oxidative phosphorylation, and 3) ARF6 protects mitochondria from oxidative injury. ARF6 may moreover promote cancer invasion and metastasis, and also acidosis and immune checkpoint. Therefore, the inseparable relationships and cooperation of KRAS, MYC, and ARF6 appear to result in the activation of mitochondria and the driving of ARF6-based malignancy and immune evasion. Such adverse associations are frequent in pancreatic cancer, and appear to be further enhanced by *TP53* mutations.

Video Abstract

**Supplementary Information:**

The online version contains supplementary material available at 10.1186/s12964-023-01130-3.

## Background

Mutations in the *KRAS* gene, and protein overexpression of c-MYC (referred to as MYC) and ARF6 are frequent in many types of cancers. *KRAS* mutations are well known to promote oncogenesis [1], MYC overexpression is thought to promote tumor growth [2], and ARF6 overexpression has been shown to promote invasion and metastasis [3]. Moreover, *TP53* is the most frequently mutated gene in human cancers [4]. Cancer cells with mutations and/or overexpression of these genes are intractable, and often exhibit recurrence. Pancreatic ductal adenocarcinoma (PDAC) is a typical example, in which mutations in the *KRAS* and *TP53* genes, and the overexpression of MYC and ARF6 proteins are all prevalent [5–7]. The 5-year patient survival rate of PDAC remains very low [8], and unfortunately immune checkpoint inhibitor therapy has not yet been approved for PDAC because it is not effective alone.

Here discussed are that KRAS, MYC, and ARF6 are biochemically and functionally closely related with each other in promoting cancer malignancy and immune evasion. *TP53* mutations may enhance the relationships and cooperation of these three musketeers by upregulating glucose uptake and the mevalonate pathway (MVP) activity, and by stopping the p53-mediated induction of certain miRNAs.

### Common features of *KRAS*, *MYC*, and *ARF6* mRNAs

*KRAS*, *MYC*, and *ARF6* mRNAs have the G-quadruplex (G4) structure in the 5'-untranslated region [9]. Although the G4 structures of various mRNAs demonstrate some differences, mRNAs with G4 generally require eIF4A, which is an RNA helicase that uses ATP to unwind the secondary structure of mRNA during translation [9]. Thus, an increase in cellular energy production appears to be a prerequisite for the robust expression of KRAS, MYC, and ARF6 proteins. Moreover, the eIF4A-mediated mRNA translation process can be the rate-limiting step for protein expression. Indeed, *ARF6* mRNA levels do not necessarily correlate with ARF6 protein levels in cancer cells [10]. Moreover, mutations in *TP53* are known to promote cellular glucose uptake [11]. Thus, *TP53* mutations appear to be efficient in increasing KRAS, MYC, and ARF6 protein expression via promoting glycolysis and ATP production.

### KRAS promotes MYC and ARF6 protein expression

*MYC* gene expression is downstream of RAS signaling [12]. Hence, *MYC* will be constitutively expressed in the presence of oncogenic mutations of *KRAS*. Moreover, KRAS may also promote the eIF-4A-dependent translation of *MYC* and *ARF6* mRNAs [7, 13]. In this process, KRAS first induces the expression of genes encoding transcription factors, such as TEAD3 and ETV4, which suppress expression of the *PDCD4* gene, which is translated into the negative regulator of eIF-4A [7]. Thus, *KRAS* mutations appear to cause the overexpression of MYC and ARF6 proteins in cancer cells. Moreover, growth factor receptor tyrosine kinases (RTKs) are frequently overexpressed in cancer. RAS is located downstream of RTK signaling. Thus, not only *RAS* mutations, but the overexpression of RTKs and their activation might also often cause ARF6 and MYC overexpression.

### Why is *KRAS* mutated instead of other *RAS* isoforms in cancer?

There is a marked bias among cancer types as to which genes among the *RAS* isoforms are frequently mutated [14]. *KRAS* mutations are predominant in cancer, particularly in PDAC, and 90% to 95% of PDACs have this mutation [1, 5]. *KRAS* mutations are also frequently found in colorectal cancer, in which approximately 35% have *KRAS* mutations [14]. On the other hand, *NRAS* mutations are found in 15% to 20% of melanomas, and *HRAS* mutations are found in about 10% of bladder cancers and cervical cancers [14]. Moreover, the mutational spectrum (*i.e*., which amino acid is frequently mutated) of *KRAS* and other *RAS* genes also varies substantially among tumors of different tissue origin, and among tumors of patients with different ages at diagnosis [15].

Unlike other *RAS* genes, the *KRAS* gene utilizes rare codons [16]. Thus, the amount of rare tRNAs present will be rate-limiting for KRAS protein expression. Very strong signals from RAS may induce cell death [17]. Thus, two features of *KRAS*, the G4 structure in the mRNA and the rare codons in the gene, will prevent the excessive expression of its protein product, and hence its mutations may be favored over mutations in other *RAS* genes in certain types of cancers. However, this notion still may not fully explain why *KRAS* mutations are so much more common in PDAC than in other types of cancer.

Each isoform of the RAS protein undergoes different lipid modifications through different intracellular transport pathways, and hence localize to different microdomains of the plasma membrane [18–20]. Furthermore, oncogenic RAS proteins and the wild-type RAS protein may play independent and nonredundant roles [21]. Detailed elucidation of the biological significance of these issues, including those described above, will lead to further understanding of the biology of cancer. In particular, the fact that almost all PDACs selectively use KRAS over other RAS-GTPases may be hiding some important secret in this cancer and could be a whole new key to drug development against PDAC.

### ARF6 in cancer

The ARF-GTPases appear to be the evolutionarily oldest type of small-GTPases, and play essential roles in the life of cells [22, 23]. This family of small-GTPases regulate membrane remodeling and intracellular trafficking [24]. ARF6 is the only member of the class III ARF-GTPases, and primarily regulates the recycling of plasma membrane components and certain cell surface receptors at the cell periphery [25]; and regulates cell adhesion and invasion [26], in which ARF6 appears to play essential roles in cell–matrix interactions and cell–cell interactions, as well as interactions with microenvironments and stromal cells (see later). Overexpression of the ARF6 protein is frequently seen in various types of cancers, including those of the pancreas, breast, kidney, lung, and head and neck, to be statistically correlated with poor patient survival [7, 27–31].

Like other small-GTPases, GTP-ARF6 uses effector proteins for its downstream signaling. AMAP1 (also called ASAP and DDEF1) is a major downstream effector of ARF6 [32]. Like ARF6, the AMAP1 protein can be overexpressed by *KRAS* mutations (7; see later). AMAP1 contains several protein–protein interaction modules, including an SH3 domain and proline-rich regions [32]. Via the interaction of AMAP1 with PRKD2 and EPB41L5, ARF6 signaling upregulates β1-integrins and downregulates E-cadherin, respectively [33, 34]. The upregulation of integrins and the downregulation of E-cadherin are hallmarks of epithelial-mesenchymal transition (EMT). EPB41L5 is a mesenchymal-specific protein that is induced upon EMT [35]. The occurrence of EMT in cancer cells is fundamentally involved in the promotion of invasion and metastasis, as well as in other malignancies such as fibrosis, and is closely associated with cancer cell resistance to treatments, including chemotherapy and immunotherapy [36–39]. Therefore, the ARF6-AMAP1 pathway appears to be crucial in promoting cancer malignancy in association with EMT. Intriguingly, moreover, the ARF6-AMAP1 pathway is also linked to the processes of intracellular recycling and cell surface expression of PD-L1 and carbonic anhydrase 9 (CA9), and hence may promote the onset of immune checkpoint (*i.e*., enhanced PD-L1 expression at the cell surface) and acidosis (*i.e*., enhanced CA9 expression at the cell surface), which both favor cancer immune evasion [7, 40]. In particular, with respect to resistance to immunotherapy, increasing intracellular recycling activity of PD-L1 by ARF6 can allow new PD-L1 molecules to continuously appear on the cell surface which may bind to PD-1 molecules on immune cells before these PD-L1 molecules blocked by their Abs. The ARF6-AMAP1 pathway furthermore has the ability to promote cancer radioresistance, by promoting the intracellular distribution of mitochondria ([41], see later).

### ARF6 converts growth stimulation into malignancy and immune evasion

The overexpression of RTKs is a major risk factor of cancer, as mentioned earlier. Such RTKs include epidermal growth factor receptor, HER2 (also called ERB-B2 or NEU), platelet-derived growth factor receptor (PDGFR), and vascular endothelial cell growth factor receptor. ARF6 can be activated by these RTKs, in which a guanine nucleotide exchanger (GEF) for ARF6, GEP100, directly binds to the tyrosine phosphorylation sites of RTKs [42]. Through this mechanism, ARF6 may convert growth stimulation into invasion, metastasis, and immune evasion [7, 42]. Furthermore, as RTK signaling can be linked to the overexpression of ARF6 and AMAP1 proteins via the activation of RAS (see later), the overexpression of RTKs may enhance ARF6 signaling in various ways.

RTKs are not a major risk factor of clear cell renal cell carcinoma. This type of cancer often overexpresses autotaxin, which is also known as ectonucleotide pyrophosphatase/phosphodiesterase 2, and produces lysophosphatidic acid (LPA) from lysophosphatidylcholine extracellularly. LPA activates ARF6 via G-protein-coupled receptors (GPCRs), in which Gα_12_ activated under the GPCRs employs EFA6, which is a GEF for ARF6 [30]. Through this mechanism, the overexpression of autotaxin acts as risk factors driving EMT-associated malignancy and the drug resistance of renal cancer [30].

### Unique properties of AMAP1

AMAP1 has a domain homologous to GTPase-activating proteins (GAPs) of ARF-GTPases, and demonstrates GAP activity against the class I ARF-GTPases [43]. On the other hand, AMAP1 binds stably to GTP-ARF6, but not to GDP-ARF6, via the GAP domain, regardless of the presence of Mg^2+^ [44]. Intriguingly, ASAP3, a close isoform of AMAP1, has been shown to demonstrate GAP activity against GTP-ARF6 in the presence of Ca^2+^ [45]. Thus, it is likely that AMAP1 also requires Ca^2+^ to hydrolyze GTP-ARF6, and this should be tested in the future.

### *KRAS* and *TP53* mutations cooperatively activate the ARF6-AMAP1 pathway

The ARF6-AMAP1 pathway appears to be the major target of *KRAS*/*TP53* double mutations. Gain-of function mutations of *TP53* promote ARF6 activation by RTKs [46]. In this process, increased expression of the MVP enzymes by gain-of function mutations of *TP53* [47] promotes the geranyl-geranylation of RAB11b, and geranyl-geranylated RAB11b then recruits ARF6 to the plasma membrane [46]. The gain-of function mutant-p53 was moreover shown to induce PDGFRβ in PDAC cells [48]; and PDGFRβ, when activated by ligands, activates ARF6 via GEP100 [7]. In line with this, statins, which are inhibitors of MHG-CoA reductase of the MVP and hence inhibit the production of geranyl–geranyl pyrophosphate, block ARF6 activation and inhibit cancer malignancy [46]. Furthermore, wild-type p53 may induce the expression of miRNAs, such as miR-96 and miR-182 that target *AMAP1* mRNA, and hence mutations in *TP53* cause an increase in *AMAP1* mRNA levels [49].

KRAS may also promote AMAP1 protein expression. *AMAP1* mRNA contains a 5'-terminal oligopyrimidine-like sequence, and requires eIF4E for translation [7]. KRAS signaling may activate mTORC1 [50], and mTORC1 then phosphorylates 4EBP1, releasing eIF4E from 4EBP1 [51]. Thus, *KRAS* mutations appear to promote *AMAP1* mRNA translation via enhancing mTORC1 and eIF4E [7]. As a result, collectively, ARF6 and AMAP1 proteins are both often overexpressed by *KRAS* mutations, and also by normal RAS activated by RTKs. Indeed, the pattern of AMAP1 overexpression among cancers is similar to that of ARF6 overexpression [10, 32].

### KRAS, MYC, and ARF6 cooperatively activate mitochondria

MYC is a transcriptional cofactor binding directly to DNA. Chromatin immunoprecipitation studies have suggested more than thousands of genes as direct targets of MYC binding [52, 53]. Intriguingly, several hundreds of these genes are involved in mitochondrial biosynthesis, and also mitochondrial functions, including oxidative phosphorylation (OXPHOS), which produces ATP aerobically [54, 55]. As KRAS induces MYC, mitochondria appear to be the primary target of KRAS and MYC in cancer [56].

ARF6 is also closely related to mitochondria. Because mitochondrial OXPHOS involves the production of reactive oxygen species (ROS), mitochondrial aggregation carries the risk of excessive ROS production by a ROS-induced ROS release (RIRR)-like mechanism [57]. Binding of the mitochondrial motor proteins RhoT1 and TRAK2 promotes retrograde mitochondrial transport [58, 59], which may lead to the accumulation of mitochondria near the nucleus. On the other hand, the ARF6-AMAP1 pathway, when activated, promotes the recycling of β1-integrin and its localization to focal adhesions via PRKD2, which in turn promotes the recruitment of integrin-linked kinase (ILK) to focal adhesions, and ILK then inhibits the RhoT1-TRAK2 association [41]. Through this mechanism, ARF6 may facilitate the forward transport and spatial distribution of mitochondria within the cell, and avoid RIRR-based oxidative injury of mitochondria [41]. In line with this, blocking the ARF6-AMAP1 pathway increased the oxidative stress of cancer cells, in association with substantial mitigation of the radioresistance [41].

The ARF6-mitochondria link appears to be important for cancer cell invasion and metastasis. The ARF6-AMAP1 pathway promotes the invasion and metastasis of cancer cells, whereas cell invasion often involves cells entering physically narrow pathways, in which mitochondria tend to be densely accumulated. Cancer cells may moreover encounter an oxygen-rich environment during invasion and metastasis, which increases mitochondrial OXPHOS, which in turn increases the risk of RIRR. Intriguingly, moreover, the link between ARF6 and mitochondria only occurs in cell invasion, but not in two-dimensional cell migration [41]. Therefore, the link between ARF6 and mitochondria appears to protect mitochondria from oxidative injury, specifically during invasion and metastasis.

**Fig. 1:**
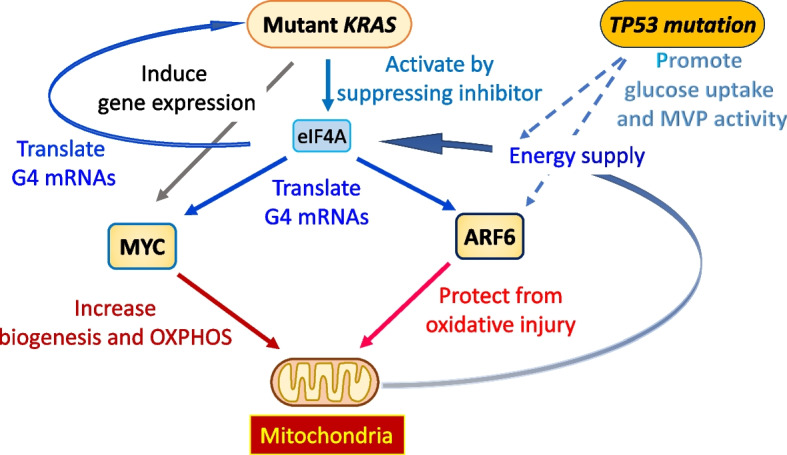
Inseparable relationships and cooperation of KRAS, MYC, and ARF6 in cancer. Mutant *KRAS* induces *MYC* gene expression and promotes *MYC* and *ARF6* G4-mRNA translation via increasing eIF4A activity. MYC promotes mitochondrial biogenesis and OXPHOS, whereas ARF6 protects mitochondria from oxidative injury by promoting anterograde trafficking of mitochondria which is linked to the activation of integrin recycling and cell invasion by ARF6. Mitochondria generate ATP by OXPHOS, which may promote eIF4A activity, which in turn promotes translation of *MYC* and *ARF6* mRNAs. *TP53* mutations enhance glucose uptake, resulting in increased anaerobic ATP production by glycolysis to facilitate the translation of G4 mRNAs even under hypoxia (*i.e.*, low mitochondrial OXPHOS). Enhanced glucose uptake may also fuel mitochondrial metabolism, including OXPHOS. *TP53* mutations enhance ARF6 activation and signaling via activating MVP and stopping expression of miRNAs that target *AMAP1* mRNA (see Fig. 2)

Collectively, as KRAS and MYC activate mitochondria, KRAS activates ARF6, and ARF6 protects mitochondria, it is likely that KRAS, MYC, and ARF6 cooperatively control mitochondria in their biogenesis, metabolism, and integrity, which are all essential for cancer growth and survival; and, again, these events are likely to be further strengthened when *TP53* is mutated (Fig. 1).

## Conclusions

**Fig. 2:**
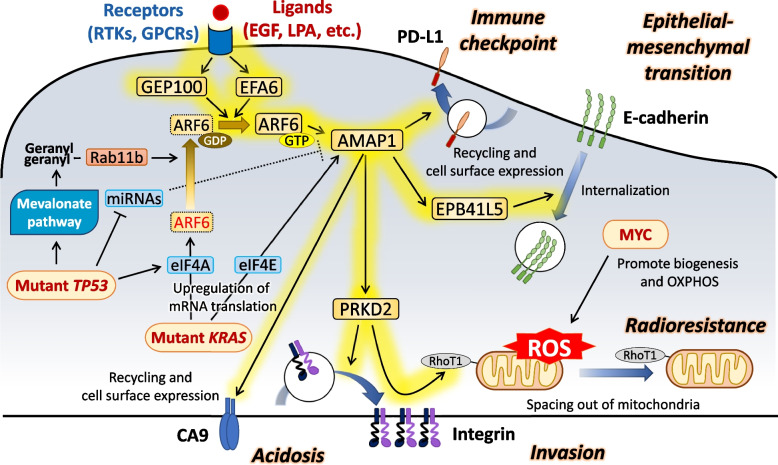
KRAS, TP53, and MYC cooperatively drive ARF6-mediated cancer malignancy and immune evasion, accompanied with increased mitochondrial activity. Activation of ARF6 by growth factors or LPA triggers a series of intracellular signaling pathways via its downstream effector, AMAP1, that promote cancer cell invasion and metastasis, immune evasion, acidosis, and oxidative/radio-resistance, respectively, and play critical roles in immune evasion and therapeutic resistance (see text for details). ARF6, KRAS, and MYC are inseparable from each other at the molecular level in promoting cancer malignancy and immune evasion. *ARF6, KRAS* and *MYC* mRNAs have the G4 structure and require high eIF4A activity for their translation. Thus, the mRNA G4 structure and eIF4A are promising targets to defeat these three evil musketeers in cancer drug development

Here I discussed the mutually inseparable relationships and cooperation of KRAS, MYC, and ARF6 in cancer malignancy and immune evasion. The molecular bases of their interrelationships are the common usage of G4 in mRNAs, and the promotion of MYC and ARF6 expression by KRAS. Their relationships and cooperation may be strengthened when *TP53* is mutated, in which the *TP53* mutation promotes G4 translation by promoting energy production, assists ARF6 activation, and increases *AMAP1* mRNA levels. The end result of this cooperation appears to be the promotion of ARF6-based cancer invasion, metastasis, acidosis, radioresistance, and immune evasion, accompanied with increased mitochondrial activity (Fig. 2).

Pharmacological inhibitors, such as the eIF4A inhibitor silvestrol, which blocks *ARF6* and *MYC* mRNA translation, and the MVP inhibitor statins, which block ARF6 activation, can effectively mitigate ARF6-based malignancies and treatment resistance [7, 40, 41, 46]. Consequently, the combination of an anti-PD-1 antibody with silvestrol very effectively blocked PDAC growth in a KPC mouse model [13]. G4 structures are also found in many human infectious microorganisms, including the malaria parasite and SARS-COV-2 virus. Thus, the development of drugs that target the G4 structure and eIF4A is now very active worldwide. Moreover, certain types of *KRAS* mutations are now known to be druggable [60, 61].

Lastly, cancer patients often have circulatory disturbances [62–65], which may affect peripheral blood mononuclear cells and hence impair immunity [66]. Immunity is essential not only in immunotherapy but also in chemotherapy-based cancer treatments [67]. Thus, as we have discussed recently [68], improving circulatory problems might be a prerequisite for effective cancer therapeutics in many cases, including those targeting KRAS and G4.

## Data Availability

Not applicable.

## Abbreviations

CA9: Carbonic anhydrase 9
EMT: Epithelial-mesenchymal transition
GAP: GTPase-activating protein
GEF: Guanine nucleotide exchange factor
ILK: Integrin-linked kinase
G4: G-quadruplex structure
LPA: Lysophosphatidic acid
PDAC: Pancreatic ductal adenocarcinoma
PDGFR: Platelet-derived growth factor receptor
ROS: Reactive oxygen species
RTK: Receptor tyrosine kinase
